# Pellagra

**Published:** 1909-08-28

**Authors:** 


### Medicine.

### PELLAGRA.

It would appear from a very complete and able article by Dr. Thayer in the *Johns Hopkins Hospital Bulletin** that Pellagra has definitely made its appearance in Maryland. This disease has lately attracted a good deal of attention in the United States, owing to the considerable number of cases recently reported from the Southern States: in South Carolina it seems clear that pellagra is well established, and has been endemic for, at the least, eighteen years; and Dr. Thayer is convinced that he has encountered two typical cases in the Northern State wherein he works. Originally described in Spain, more than one hundred and fifty years ago, it was soon also recognised in Italy and France. In the latter country it was very prevalent during the first half of the nineteenth century, but is now almost unknown; in the former it is still rampant, as is shown by the fact that there are in that kingdom twenty-two special hospitals for the reception of patients suffering from it. Pellagra exists also in Portugal, the Tyrol, Dalmatia, Greece, Bosnia, and other provinces of Eastern Europe; in Egypt, Mexico, Brazil, the Argentine, Barbados, and New Caledonia.

### Theories of Ætiology.

From the observed fact that in Italy the disease afflicts almost solely the peasant population, who live very largely on maize, a belief long ago arose that pellagra is a consequence of the ingestion of mouldy or otherwise spoiled corn contaminated by some organism or organisms which, either through their own toxic products or through poisons produced by the decomposition of the maize, or both, give rise to the symptoms. This question has been seriously and carefully debated in Italy, but nothing has been certainly proved either way, and it is by no means settled that maize-eating has anything to do with the origin of pellagra. Nevertheless it is worthy of remark that active preventive measures in Italy are directed mainly to the exclusion of spoiled corn from the dietary of the agricultural classes by the introduction of artificial desiccating machinery, of public store houses, of corn exchanges, and of rural bakeries whence wheaten bread is supplied to the peasants instead of bread made from maize. It is said that the ravages of pellagra are already being considerably curtailed by these measures: considering that in certain provinces the actual mortality rate has varied in recent years from 18 to 36 per 100,000 per annum, and that the mortality rate is reckoned at no more than 2½ per cent. of the cases, it is clear that there is ample room for further improvement.

### Seasonal Incidence of the Disease.

A very peculiar feature of the disease is its seasonal incidence. The onset of symptoms is nearly always in the spring, which during the ensuing summer tend to improvement; relapse is not uncommon about October, but by the New Year the patient may be perfectly recovered, only to begin a new attack when the spring again comes round. Two forms, acute and chronic, are described: the former runs a rapid course, marked especially by delirium, fever, and uncontrollable diarrhœa, and ends fatally in a few weeks; the latter may last as long as 25 years, though as a rule each year sees the attack a little more severe and the patient a little more exhausted than before.

### Symptoms.

The symptoms of pellagra are described in three divisions, according as they affect (1) the alimentary tract, (2) the skin, (3) the nervous system. Of the first, nausea and dyspepsia are frequent, diarrhœa is especially prominent, and vomiting is by no means uncommon. An aphthous stomatitis renders eating and swallowing extremely painful, and salivation may be most distressing. The cutaneous symptoms, however, are perhaps the most constant and obvious. On the backs of the hands, and in those who go barefoot on the dorsa of the feet, appears a brilliant red symmetrical erythema, which may extend as low as the proximal interphalangeal joints and as high as a point just above the wrists. The skin soon becomes dry and scaly, and then exfoliates, leaving cracks and fissures and a raw red surface: sometimes large bullæ form, containing serum or pus, or even blood. The nervous symptoms are also very constantly found. Disturbances of sensation, spastic paralyses, and weakness of sphincters may be found; the deep reflexes are generally increased, especially in the lower limbs. Vertigo is common.

The symptoms point, as a rule, to varied and continued spinal lesions; and in the spinal cord itself the posterior columns, the posterior nerve roots, and the lateral columns have all been described as sclerotic. Mental phenomena are frequently associated: confusion, weakness of will, depression, and disorientation as to time and place may go on to melancholia with suicidal tendencies, or in some cases to emotional and maniacal states. Finally, dementia is a common termination of chronic and recurrent cases. It is estimated that about a tenth of the patients become permanently insane. Hallucinations of sight and hearing, mutism, delusions of persecution, and so on, are fairly frequent.

### The Disease in America.

Such is the disease whose appearance in the United States is attracting the attention of clinicians there. It is but seven years since the first case was described, but when once the discovery had been made it came to light that un-

* July, 1909.

recognised cases had been under treatment for at least eleven years. It is somewhat extraordinary too, that, whereas in Europe only the poorest of the poor are attacked, in America there have been numerous cases among well-to-do people, several of whom have rapidly ended in a fatal issue. Although maize is grown in the United States much more extensively than in Britain, it is difficult to suppose that mouldy grain enters into the dietary of such patients; and it is the negro population which consumes by far the larger part of the maize crop. The descriptions given of the two cases which Dr. Thayer has had the opportunity of seeing seem to establish quite clearly the diagnosis; and his warning to American practitioners to look out for the disease is evidently timely. Since visitors from the States are present in England in very considerable numbers at this time of year, and since the maize theory of pellagra is yet unproved, it will be quite worth while for medical men in this country also not to forget that such a syndrome exists, though it is, of course, in the highest degree unlikely that any cases of it will come to light.

Thyroid Extract in the Treatment.

In the first case of which the details are given there seemed to be symptoms of hypothyroidism superadded to those of pellagra, and accordingly thyroid extract was administered in 2-grain doses thrice a day. The improvement was marked and immediate, and not only did the signs of hypothyroidism rapidly pass away, but the pellagra also in time disappeared, and has not returned. This patient is still, after four years, taking thyroid extract regularly, as she finds that to omit it for a week is followed by the commencing reappearance of nervous symptoms. The question whether the thyroid gland had any direct therapeutic influence on the pellagra is left open, in view of the fact that in several recorded cases it has produced no effect at all. All that the author cares to hazard is the suggestion that possibly a condition of hypothyroidism renders the individual unduly susceptible to the unknown cause of pellagra.

